# Pediatric Exercise Testing: Value and Implications of Peak Oxygen Uptake

**DOI:** 10.3390/children4010006

**Published:** 2017-01-24

**Authors:** Paolo T. Pianosi, Robert I. Liem, Robert G. McMurray, Frank J. Cerny, Bareket Falk, Han C. G. Kemper

**Affiliations:** 1 Department of Pediatric and Adolescent Medicine, Mayo Clinic, 200 First St. SW, Rochester, MN 55905, USA; 2 Division of Hematology, Oncology & Stem Cell Transplant, Ann & Robert H. Lurie Children’s Hospital, Chicago, IL 60611, USA; rliem@luriechildrens.org; 3 Department of Nutrition and Exercise & Sport Science, University of North Carolina, Chapel Hill, NC 27514, USA; mcmaro21@gmail.com; 4 Women’s and Children’s Hospital of Buffalo, Department of Pediatrics and Exercise and Nutrition Sciences, University at Buffalo, Buffalo, NY 14214-8028, USA; Frank.cerny@verizon.net; 5 Faculty of Applied Health Sciences, Brock University, St. Catharines, ON L2S 3A1, Canada; bfalk@brocku.ca; 6 EMGO + Institute for Health and Care Research, VU University Medical Center, 1081 Amsterdam, The Netherlands; hancgkemper@upcmail.nl

**Keywords:** cardiopulmonary exercise testing, peak oxygen uptake, aerobic fitness

## Abstract

Peak oxygen uptake (peak$\dot{V}O_{2}$) measured by clinical exercise testing is the benchmark for aerobic fitness. Aerobic fitness, estimated from maximal treadmill exercise, is a predictor of mortality in adults. Peak$\dot{V}O_{2}$ was shown to predict longevity in patients aged 7–35 years with cystic fibrosis over 25 years ago. A surge of exercise studies in young adults with congenital heart disease over the past decade has revealed significant prognostic information. Three years ago, the first clinical trial in children with pulmonary arterial hypertension used peak$\dot{V}O_{2}$ as an endpoint that likewise delivered clinically relevant data. Cardiopulmonary exercise testing provides clinicians with biomarkers and clinical outcomes, and researchers with novel insights into fundamental biological mechanisms reflecting an integrated physiological response hidden at rest. Momentum from these pioneering observations in multiple disease states should impel clinicians to employ similar methods in other patient populations; e.g., sickle cell disease. Advances in pediatric exercise science will elucidate new pathways that may identify novel biomarkers. Our initial aim of this essay is to highlight the clinical relevance of exercise testing to determine peak$\dot{V}O_{2}$, and thereby convince clinicians of its merit, stimulating future clinical investigators to broaden the application of exercise testing in pediatrics.

## 1. Introduction

Aerobic fitness refers to the body’s ability to transport oxygen from the environment and utilize it in working muscle. It is well accepted as a health-related and health-determining trait since Blair et al. demonstrated more than 25 years ago that higher aerobic fitness measured during an incremental treadmill exercise test was associated with reduced all-cause mortality in adults [1]. Exercise testing has proven to be an invaluable tool in research and practice, because it yields otherwise unobtainable physiologic information on clinical manifestations, adaptations, and compensatory strategies, in a variety of disorders. There are many terms used to convey this concept of aerobic fitness, but for the purposes of this manuscript, terminology will be confined to peak oxygen uptake (peak$\dot{V}O_{2}$) or aerobic fitness.

Increased attention to the health and fitness of toddlers, children, and adolescents, and to the concept of pediatric origins of adult disease, have brought exercise testing to the forefront as a clinical and research tool; in turn, leading to the imperative of standardizing methodologies. Therapeutic advances have created a growing number of childhood survivors of a wide range of conditions, including preterm birth, congenital heart disease, pulmonary hypertension, cystic fibrosis, sickle cell disease, and cancer. In addition, the same environmental and social factors contributing to the obesity epidemic in otherwise healthy children are at work in children with chronic disease and disability. High levels of physical activity are (or were) the norm in children, and a child’s inability to engage in physical activity is often one of the first indicators of childhood disease. We submit that peak$\dot{V}O_{2}$ is a biomarker for the development and severity of various health outcomes. Devising and investigating new medical therapies in children has always been a challenge, but exercise testing can become a benchmark or outcome measure in clinical trials.

## 2. Discussion

### 2.1. Peak Oxygen Uptake (Peak$\dot{V}O_{2}$) in Children and Adolescents

Maximal exercise testing is essential for assessing peak$\dot{V}O_{2}$, and provides information on the function of respiratory, circulatory, neuromuscular, blood, and metabolic systems, as well as limits of exercise tolerance. Physiological limitations become apparent when working muscle can no longer sustain the task because the muscle lacks sufficient metabolic capacity, the cardiovascular system cannot deliver sufficient oxygen, or the respiratory system cannot provide sufficient oxygen transfer or carbon dioxide removal, resulting in intolerable acidosis. Ultimately, these signals are interpreted at the cerebral level, prompting the individual to cease exercise, manifested by inability to continue the test despite internal motivation or external urging. Therefore, the measurement of peak$\dot{V}O_{2}$ is the consummate measure of cardio-pulmonary and muscle-metabolic function, and thus is useful in the diagnosis, management, and prognosis of disease. It is most readily determined with a progressive incremental exercise test to voluntary exhaustion. The end point of the test should be maximal exertion [2,3] or the limit of exercise tolerance. Whereas it is simple for the purposes of this paper to define peak$\dot{V}O_{2}$, clear criteria or guidelines to indicate the attainment of a gold standard measure remain elusive [4,5], rendering comparisons between studies or individuals a not-insurmountable challenge. A major issue in conducting maximal exercise tests is defining the end point(s) for achieving peak$\dot{V}O_{2}$ in pediatric subjects. In adults, achieving a plateau in oxygen uptake, (i.e., no increase in $\dot{V}O_{2}$ with an increase in work) has been proposed as the ultimate indicator of achieving maximum $\dot{V}O_{2}$ or true $\dot{V}O_{2}$max [6]. However, children often do not attain a plateau in oxygen uptake, despite indicating that they have reached their limit of tolerance [5,7]. Some would argue that mere determination of the so-called “anaerobic” or “ventilatory” threshold is simpler and therefore preferable, but this parameter is indeterminate in ~20% of subjects [8], and it can have a large range of inter-reviewer variability, rendering it unsuitable for clinical use [9].

The attainment of peak$\dot{V}O_{2}$ relies on two components: central and peripheral. The central component is responsible for the bulk convection of oxygen from the ambient environment to the working muscle. The equally important peripheral component is determined by how effectively exercising muscle utilizes substrate for energy production, one index of which is arteriovenous oxygen content difference. Reported exercise tolerance is affected by many factors that are virtually impossible to identify and control for completely (e.g., climatic factors, specific test modality, etc.). Exercise tolerance and termination during an incremental test to maximal effort is influenced by the pre-frontal cortex [10,11]—a motivational factor virtually impossible to estimate or measure. Moreover, the underlying presumption that peak$\dot{V}O_{2}$ is a measure of global cardiac and pulmonary function essentially ignores the muscle component, though muscle function may play a greater role than cardio-respiratory factors in determining overall aerobic fitness in certain clinical populations. Sadly, our understanding of peripheral muscle performance in determining overall aerobic exercise capacity is wanting, because it is the most difficult component to study. Newer techniques such as near-infrared spectroscopy may shed light on this “black box” [12]. 

One must inject a cautionary note when peak$\dot{V}O_{2}$ is estimated from sub-maximal field tests or sub-maximal cycling or treadmill tests, as such methods do not maximally stress the body’s systems, and typically estimate peak$\dot{V}O_{2}$ based on a standardized algorithm. In general, these approaches are characterized by large variability, and therefore less reliability, validity, and robustness. That is not to say that data obtained from submaximal exercise testing are without merit [13]. In certain patient populations, rates of change (slope) of particular variables during incremental exercise have prognostic value [14,15]. Other parameters obtained during sub-maximal exercise, such as gas exchange or anaerobic threshold [9,16] terms and oxygen uptake efficiency slope [17] are purported to be useful, though each has unique problems with determination or interpretation.

### 2.2. Peak$\dot{V}O_{2}$ and Health Outcomes in Children

#### 2.2.1. Cystic Fibrosis as Paradigm

There is a growing body of literature on the clinical significance of exercise testing and peak$\dot{V}O_{2}$ in children with chronic disease. Nixon et al. first reported that peak$\dot{V}O_{2}$ was a strong predictor of survival in young cystic fibrosis (CF) patients [18], an observation that was later replicated [19]. Indeed, peak$\dot{V}O_{2}$ has supplanted the previous standard of forced expiratory volume in one second (FEV_1_) in predicting mortality in CF patients awaiting lung transplantation [20]. In patients with CF, certain classes of cystic fibrosis transmembrane conductance regulator (CFTR) mutations (I and II) are associated with more severe disease status, and reduced peak$\dot{V}O_{2}$ is also associated with these classes of mutations [21], showing a link between genotype and a biomarker (peak$\dot{V}O_{2}$). The importance of genotype-specific treatment for CF was recently demonstrated in a clinical trial of mutation-specific therapy for patients with the G551-D CFTR mutation. Not only did this therapy result in sustained improvement in FEV_1_, but also in weight gain and overall enhanced health-related quality of life as well [22]. As the consummate test of cardiopulmonary function, the maximal exercise test is uniquely capable to capture these inter-related measures of treatment benefit(s). Furthermore, change in peak$\dot{V}O_{2}$ over time is more useful as a prognostic marker than the commonly-used longitudinal decline of FEV_1_ [19]. Information that can be gained from exercise tests eventually prompted the adoption of a position statement on the value of exercise testing in cystic fibrosis [23].

#### 2.2.2. Congenital Heart Disease

There is increasing survival among children with congenital heart defects, prompting the development of specialized clinics for adults with congenital heart disease. In the past decade, reports on exercise testing in this population have proven invaluable for risk stratification [24]. Peak$\dot{V}O_{2}$ and heart rate reserve were shown to provide the greatest predictive information for prognosis, after adjusting for age, cyanosis, and need for negative inotropes [14,15,25]. Many such patients report exertional dyspnea, and one of the most interesting data bytes emerging from exercise studies addresses the tedious clinical conundrum “is it the heart or is it the lung?”. Hyperventilation during exercise—defined as an exaggerated slope of ventilation vs. CO_2_ production—is ubiquitous in adults with various types of congenital heart disease, and was the strongest predictor of mortality in non-cyanotic patients [15]. In some—particularly those with Fallot’s tetralogy—hyperventilation may result from maldistribution of pulmonary blood flow related to residual pulmonary stenosis. Balloon angioplasty in such patients improved not only ventilatory efficiency, but also peak$\dot{V}O_{2}$ due to improved forward stroke volume [26]. Thus, it is both heart *and* lungs!

Peak$\dot{V}O_{2}$ is an independent predictor of hospitalization or mortality in patients with surgically repaired tetralogy of Fallot [27], and in patients who have undergone atrial switch procedures for transposition of the great vessels [28]. The latter patients are now adults, and lingering concerns about long-term sustainability of the right ventricle acting as the systemic ventricle are being addressed by exercise testing [29,30]. While much work still needs to be done, exercise testing—particularly with the measurement of stroke volume—permits identification of those who benefit from exercise training, and by default, those in whom pharmacologic therapy may be indicated [31,32]. 

The Fontan procedure has been lifesaving for many infants with previously lethal forms of congenital heart disease. Measurement of peak$\dot{V}O_{2}$ has not delivered the promise of a tangible outcome measure, perhaps because maximal effort as described above is difficult to achieve in these patients [33,34]. Nevertheless, reports of sub-maximal exercise results are providing insights and laying a foundation for future investigation or interventions. Based on experience in other congenital heart diseases, exercise testing may well yield prognostic data as well.

#### 2.2.3. Pulmonary Hypertension

Peak$\dot{V}O_{2}$ has prognostic value in children and adolescents with pulmonary hypertension, as reported by the Task Force on End Points and Clinical Trial Design [35]. The first study using peak$\dot{V}O_{2}$ as an outcome measure in children with pulmonary arterial hypertension demonstrated improved oxygen delivery and exercise capacity in children treated with a moderate and high dose of sildenafil [36]. The finding that a low dose did not improve peak$\dot{V}O_{2}$ while a high dose was associated with higher mortality triggered a US Food and Drug Administration (FDA) black box warning regarding use of sildenafil for pulmonary hypertension in pediatrics, the wisdom of which has been questioned [37].

#### 2.2.4. Sickle Cell Disease

The use of exercise testing to assess cardiopulmonary fitness in sickle cell anemia (SCA) has been relatively sparse compared to other conditions, despite initial reports in this population over 30 years ago [38,39,40]. Measurement of peak$\dot{V}O_{2}$ in children and adults with SCA has been limited to date by concerns regarding the safety of pushing individuals with SCA to voluntary exhaustion during exercise testing. The pathophysiology of SCA is characterized by accelerated red blood cell breakdown, microvascular occlusion, and a pro-inflammatory state—all of which may lead to acute pain episodes as well as acute and chronic organ injury over the lifespan. Although triggers for acute pain episodes vary but may include high intensity exercise, the safety of maximal exercise testing in children with SCA has now been demonstrated [41,42].

Peak$\dot{V}O_{2}$ has been found to be significantly lower (up to 30% in recent studies) in children with SCA, when compared to matched controls [41]. This finding is not surprising, given the known impact of the disease on physical functioning, as assessed by health-related quality of life surveys or surrogates for peak$\dot{V}O_{2}$, such as the 6-min walk distance [43,44]. Compared to what has been established for other conditions, less is known about the clinical significance or prognostic implications of low peak$\dot{V}O_{2}$ in SCA. Given the complexity and multi-organ nature of the disease, factors beyond anemia likely contribute to limitations in peak$\dot{V}O_{2}$ in SCA. Cardiopulmonary complications such as pulmonary vascular disease and diastolic dysfunction are associated with lower peak$\dot{V}O_{2}$ and other measures of exercise capacity in adults with SCA [45,46]. Recent data suggest low peak$\dot{V}O_{2}$ may also be associated with a greater inflammatory response to exercise, as defined by an increase in levels of vascular cell adhesion molecule [47]. However, the utility of peak$\dot{V}O_{2}$ to predict clinically relevant endpoints such as pain episodes, hospitalizations, or mortality has not yet been studied. In many ways, SCA represents an ideal disease model to examine whether interventions to improve peak$\dot{V}O_{2}$ could have a biological impact on inflammation and vascular health, and therefore lead to clinical benefits.

### 2.3. Need for Reference Values

There is no agreement as to what constitutes low peak$\dot{V}O_{2}$ in children. Establishment of clinical thresholds or recommended levels of health outcomes in children and adolescents typically has two requirements: establishing age-, sex-, and possibly ethnic-based normative values; and determining specific levels which affect health and disease outcome. The first requirement involves the understanding of the general developmental pattern in the trait of interest (e.g., age- and sex-associated variation). Indeed, the lack of agreement on cut-off values for children may be related to the age-, sex-, and maturity-related changes in peak$\dot{V}O_{2}$. The developmental pattern of peak$\dot{V}O_{2}$ has been well-studied in the classic cross-sectional studies of Astrand [6], as well as more recent longitudinal studies [48,49], and has been extensively reviewed [50,51,52]. Absolute peak$\dot{V}O_{2}$ (mL/min) of girls is typically similar to or slightly lower than that that of boys until puberty, when peak$\dot{V}O_{2}$ of girls reaches a plateau while that of boys rises further. Expressed relative to body mass (mL/kg/min), boys’ peak$\dot{V}O_{2}$ remains constant through adolescence, while that of girls decreases after puberty [2].

The second requirement of the establishment of clinical thresholds is to determine a specific level that adversely impacts the risk of health or disease outcome. Some health-related outcomes have well-accepted clinical cut-points (e.g., cholesterol, glucose, etc.). Based on an increased risk of metabolic syndrome, McMurray et al. suggested that low peak$\dot{V}O_{2}$ is <37 mL/kg/min for girls and <42 mL/kg/min for boys [53]; whereas others simply used receiver operating curves to identify the lowest quartile as the cut-off for reduced peak$\dot{V}O_{2}$ [54]. The problem with choosing an absolute cut-off lies in the fact that some studies report lower values for normal, healthy children [55]. One could argue that defining “normal” based on the average and deviation from the average is in itself problematic, since “normal” may in fact be below an acceptable level based on risk factors for some adverse health outcomes. Nevertheless, while normative and clinical thresholds of peak$\dot{V}O_{2}$ values are not yet widely established, a working basis is extant.

#### Importance of Data Harmonization

The main obstacles to using the physiologic response to exercise as an outcome in clinical trials are: (1) the lack of well-accepted and precise definitions of exercise outcome variables in children; (2) the lack of mechanisms to support the sharing of exercise-derived data among separate institutions; and (3) the lack of commonly-accepted protocols for exercise testing and physical activity assessment in the laboratory. We submit that cardiopulmonary exercise testing and measurement of peak$\dot{V}O_{2}$—despite the inherent challenges—is essential in pediatric medicine, and therefore merits our best, concerted efforts. Large studies using the physiologic response to exercise in children as an outcome measure are needed to address feasibility concerns of single center trials and to accrue generalizable data. Such an effort will be possible when harmonized protocols for testing, acquisition, and reporting are established, permitting one to measure peak$\dot{V}O_{2}$ in many centers. Multicenter trials introduce another set of feasibility concerns that must be addressed—a challenge best tackled by data harmonization, which can reduce or minimize these obstacles. It will not only facilitate the large-scale studies required to advance pediatric care, but will also ultimately lead to the establishment of true, nationally representative, normative data for children’s physiological response to exercise. The Data Harmonization in Exercise Data study group has identified a strategy and direction for achieving data harmonization which can be accomplished by carefully examining exercise data as well as data collection and reporting protocols for peak$\dot{V}O_{2}$ data from multiple institutions [56].

## 3. Future Direction

Much of what is known about exercise physiology has been derived from studies done in adults, yet every pediatrician knows that lessons learned from research in adults do not necessarily apply to children or even to adolescents. There is a growing body of literature defining the state-of-the-art of pediatric exercise medicine, though progress has been slow. This paper advocates the advancement of exercise science in general—and exercise testing in particular—in pediatrics by providing a rationale based on demonstrated prognostic value of data so obtained. Our focus has been on peak oxygen uptake (the conventional measure of aerobic fitness), since peak$\dot{V}O_{2}$ is the consummate test of cardiopulmonary and muscle function, and has already proven itself as a biomarker for longevity. Fundamentally, one need only consider the Barker hypothesis and developmental origins of health and disease to grasp this. Inactivity is a key risk factor in the development of most chronic diseases and cancers, and inactivity typically results in low peak$\dot{V}O_{2}$. This is alarming, given that physical activity patterns track from childhood into adulthood [57,58,59]. It does not require a leap of faith to envision peak$\dot{V}O_{2}$ as a valid biomarker in health or disease during the developmental years that awaits exploration. This essay highlighted common diseases where it has been found to be particularly useful, but progress has been impeded by under-appreciation of the merits of clinical exercise testing, and perhaps over-estimation of barriers to its conduct. One hopes this overview foments change in such attitudes.
